# The relation of meiotic behaviour to hybridity, polyploidy and apomixis in the *Ranunculus auricomus* complex (Ranunculaceae)

**DOI:** 10.1186/s12870-020-02654-3

**Published:** 2020-11-17

**Authors:** Birthe H. Barke, Kevin Karbstein, Mareike Daubert, Elvira Hörandl

**Affiliations:** 1 Department of Systematics, Biodiversity and Evolution of Plants, Albrecht-von-Haller Institute for Plant Sciences, University of Goettingen, Untere Karspuele 2, D-37073 Goettingen, Germany; 2 Present Address: Carl von Ossietzky University, Institute of Biology and Environmental Sciences, Carl von Ossietzky Straße 9-11, D-26129 Oldenburg, Germany

**Keywords:** Developmental biology, Gametophytic apomixis, Hybrid, Meiosis, PMC, Polyploidy, *Ranunculus*

## Abstract

**Background:**

Hybridization and polyploidization are powerful evolutionary factors that are associated with manifold developmental changes in plants such as irregular progression of meiosis and sporogenesis. The emergence of apomixis, which is asexual reproduction via seeds, is supposed to be connected to these factors and was often regarded as an escape from hybrid sterility. However, the functional trigger of apomixis is still unclear. Recently formed di- and polyploid *Ranunculus* hybrids, as well as their parental species were analysed for their modes of mega- and microsporogenesis by microscopy. Chromosomal configurations during male meiosis were screened for abnormalities. Meiotic and developmental abnormalities were documented qualitatively and collected quantitatively for statistical evaluations.

**Results:**

Allopolyploids showed significantly higher frequencies of erroneous microsporogenesis than homoploid hybrid plants. Among diploids, F_2_ hybrids had significantly more disturbed meiosis than F_1_ hybrids and parental plants. Chromosomal aberrations included laggard chromosomes, chromatin bridges and disoriented spindle activities. Failure of megasporogenesis appeared to be much more frequent in than of microsporogenesis is correlated to apomixis onset.

**Conclusions:**

Results suggest diverging selective pressures on female and male sporogenesis, with only minor effects of hybridity on microsporogenesis, but fatal effects on the course of megasporogenesis. Hence, pollen development continues without major alterations, while selection will favour apomixis as alternative to the female meiotic pathway. Relation of investigated errors of megasporogenesis with the observed occurrence of apospory in *Ranunculus* hybrids identifies disturbed female meiosis as potential elicitor of apomixis in order to rescue these plants from hybrid sterility. Male meiotic disturbance appears to be stronger in neopolyploids than in homoploid hybrids, while disturbances of megasporogenesis were not ploidy-dependent.

## Background

In all eukaryotic organisms, meiosis is the core of sexual reproduction, which ensures recombination and thus evolution and speciation [e.g. [1]]. This type of cell division manages to half the chromosome number of a diploid organism in order to produce four haploid gametes. Meiosis requires one step of DNA replication followed by two chromosome segregation processes [meiosis I and II, [2]]. The most important and therefore tightly controlled part of the whole mechanism is the formation of crossing overs among homologous chromosomes facilitating genetic recombination during meiosis I [3]. Exact chromosome segregation is strictly required since unbalanced gamete formation can lead to cell death, sterility or aneuploidy [4].

Interspecific hybridization is a frequent phenomenon in plants [5], which results either in offspring with a doubled chromosome number (allopolyploids) or in diploid hybrids [homoploids, [6, 7]]. Hybridization creates a versatile range of hybrids with each different genotypes and divergent fitness [5]. Natural hybrids generally have a negative connotation and are even termed as “hopeful monsters” because of their reduced fitness [8]. This means that these plants are often inviable or sterile, while suffering from a lack of mating partners due to isolation e.g. through divergent ploidy levels [5, 8]. The strongest effects of hybridization on plant fertility are usually found in F_1_ hybrids [8, 9]. The combination of divergent chromosomes can oblige lack of homolog pairing and segregation at meiosis, depending on the differences between parental species. Strong discrepancies are assumed to result in deleterious consequences for sporogenesis, gametophyte development and gamete formation [7, 10, 11].

However, fertility of plant hybrids is highly variable, and eventually subsequent generations can establish novel evolutionary lineages [5]. Historically, homoploid hybrid speciation was assumed as rarely arising phenomenon [e.g. [5, 12]] because of missing concrete identification evidence [7]. The importance of this topic among evolutionary biologists grew, while only a few cases of homoploid hybrid plants are known [13]. Best documented and described natural homoploid hybrids belong to the taxa *Helianthus*, *Senecio*, *Doronicum* and *Iris* [e.g. [14–17]]. Homoploid hybrids possess half of the chromosome set of each parent, which strongly limits reproductive isolation of these hybrids. Speciation of homoploid hybrids is unlikely because gene flow is not efficiently suppressed, as it is in allopolyploids [8, 12] but reproduction isolation can be achieved by spatial isolation, karyotype and/ or ecological divergence [7].

Polyploid plants have to organize and maintain functionality with more than two complete chromosome sets. Neopolyploids are therefore considered to be genetically and phenotypically unstable and prone to meiotic errors [10]. Such errors get less over generations because the polyploid chromosome set becomes stabilized by cytological diploidization that acts on gamete formation [18]. During diploidization genetic and chromosomal configuration is drastically restructured e.g. redundant chromosomes are eliminated and gene duplicates can get disposed or new functions can be assigned [neofunctionalization, [4, 10]]. Autopolyploids are the result of restitutional meiosis, gaining unreduced gametes that develop into plants with increased ploidy level, often via a triploid bridge [19]. In contrast, allopolyploids are not only caused by unreduced gamete formation, but additionally by a hybridization event of two species. Meiosis in autopolyploids is disturbed due to fact that such plants are equipped with more than two copies of each chromosome, which favours the emergence of homologous multivalents, while allopolyploids are commonly able to develop regular bivalents during prophase I. Nonetheless, young allopolyploid plants prevalently show meiotic mistakes as well but less frequent compared to autopolyploids [11]. Indeed, the frequency and likelihood of allopolyploids recognizing one or more homeologous pairing partners fundamentally depends on sequence divergences of the parental genomes. Difficulties in chromosome alignment and synapsis still occur on regular basis in young diploid hybrids due to the forced pairing of even homeologous partners. Overall, polyploid plants with hybrid origin tend to behave during meiosis as diploids, because the homologs derived from the same parent can form bivalents [4, 10]. This way, the problems of homeolog pairing can be avoided.

Apomixis, which is asexual seed formation, circumvents meiosis in various developmental pathways [20]. One common form of apomixis involves mitotic embryo sac (ES) development out of a somatic nucellar cell (apospory), resulting in clonal, maternal egg cells [21]. This specialized mode of reproduction is able to avoid negative effects of allopolyploidy on meiosis and is in natural populations often regarded as an escape from hybrid-caused sterility [10, 20, 22, 23]. Indeed, most apomicts are polyploids and/ or hybrids but how apomixis is triggered in natural plant populations is still under debate [23].

However, in the context of meiotic errors, apomictic reproduction seems to represent a powerful tool in saving plants from deleterious consequences like chromosome mispairing and –segregation upon hybridization and (allo-) polyploidization. In plants, apomixis only affects female development, where meiosis is difficult to observe directly. On the male side, however, no specific developmental pathways evolved in apomictic plants, and pollen is mostly meiotically reduced. Meiosis research, especially those studies including cytological investigations, is in plants traditionally done on pollen mother cells (PMCs) only because of easier observation [e.g. [2, 24, 25]]. Due to these technical reasons, only a few empirical studies are available on a possible correlation of meiosis behaviour and expression of apomixis [26, 27]. It is further unclear whether male meiosis phenotypes can be regarded as a predictor for female meiosis and development, when they occur in the same hermaphroditic plant.

The *Ranunculus auricomus* complex includes about 800 described species [28]. The vast majority of these species are apomictically reproducing polyploids, while a small number of species are diploid (2*n* = 16) and tetraploid (2*n* = 32) sexuals [29–33]. The sexual species, *R. notabilis*, *R. carpaticola,* and *R. cassubicifolius,* are obligate outcrossers [32, 34] and can be regarded as progenitors of the whole polyploid complex [28, 35, 36]. *R. notabilis* and the more closely related species pair *R. cassubicifolius/ carpaticola* represent two genetically and morphologically distinct lineages that separated c. 600,000 years ago [29, 35, 37–39]; all three taxa occur in geographical isolation [map in [39]]. Functional apomixis in *Ranunculus* demands effective coupling of apomeiosis and parthenogenetic egg cell generation [21]. Unsuccessful linkage of these two crucial steps towards apomictic reproduction can result in increased offspring ploidy [21, 40].

In fact, cytological analysis in the *R. auricomus* complex has been performed on either female or male sporogenesis as well as subsequent processes focusing on gametogenesis and following processes such as pollen quality determination [21, 29, 41, 42]. Reduced female fertility of F_1_ hybrids between *R. notabilis* and *R. cassubicifolius/ carpaticola* has been observed in experimental crosses [34], and apospory has been observed in F_1_ and F_2_ hybrids [32, 40, 43]. The present study provides an analysis of chromosomal behaviour in *Ranunculus* pollen mother cells (PMCs) during sporogenesis and beyond. We want to compare here disturbances of meiosis versus normal meiotic succession, without a focus on a specific stage of meiosis. This allows a comparative evaluation of development in di- and polyploid natural sexual and apomictic species as well as of two synthetic, diploid and polyploid hybrid generations that represent an intermediate phase between sexuality and apomictic reproduction. Additionally, these results are qualitatively and quantitatively compared to disturbances of megagametogenesis in di- and polyploid F_2_ hybrid plants that have shown different frequencies of apospory and asexual seed formation [40]. Aposporous initials appear in general at the end of megasporogenesis, but were neither observed at earlier meiotic stages nor in ovules without meiosis [26, 32, 40, 43]. Hence, we want to test a hypothesis that disturbances of meiosis might affect the appearance of aposporous initial cells. We expected an increase in abnormal microsporogenesis, not only within synthetic, diploid and polyploid *Ranunculus* hybrids but also in young natural polyploids with hybrid background. Results, however, suggest different meiotic behaviour in diploid versus polyploid plants, and also different selective constraints for female and male sporogenesis. We conduct here phenotypic investigations, which might give directions for future studies on molecular control mechanisms.

## Results

### Male meiosis, microsporogenesis and pollen formation

**Fig. 1:**
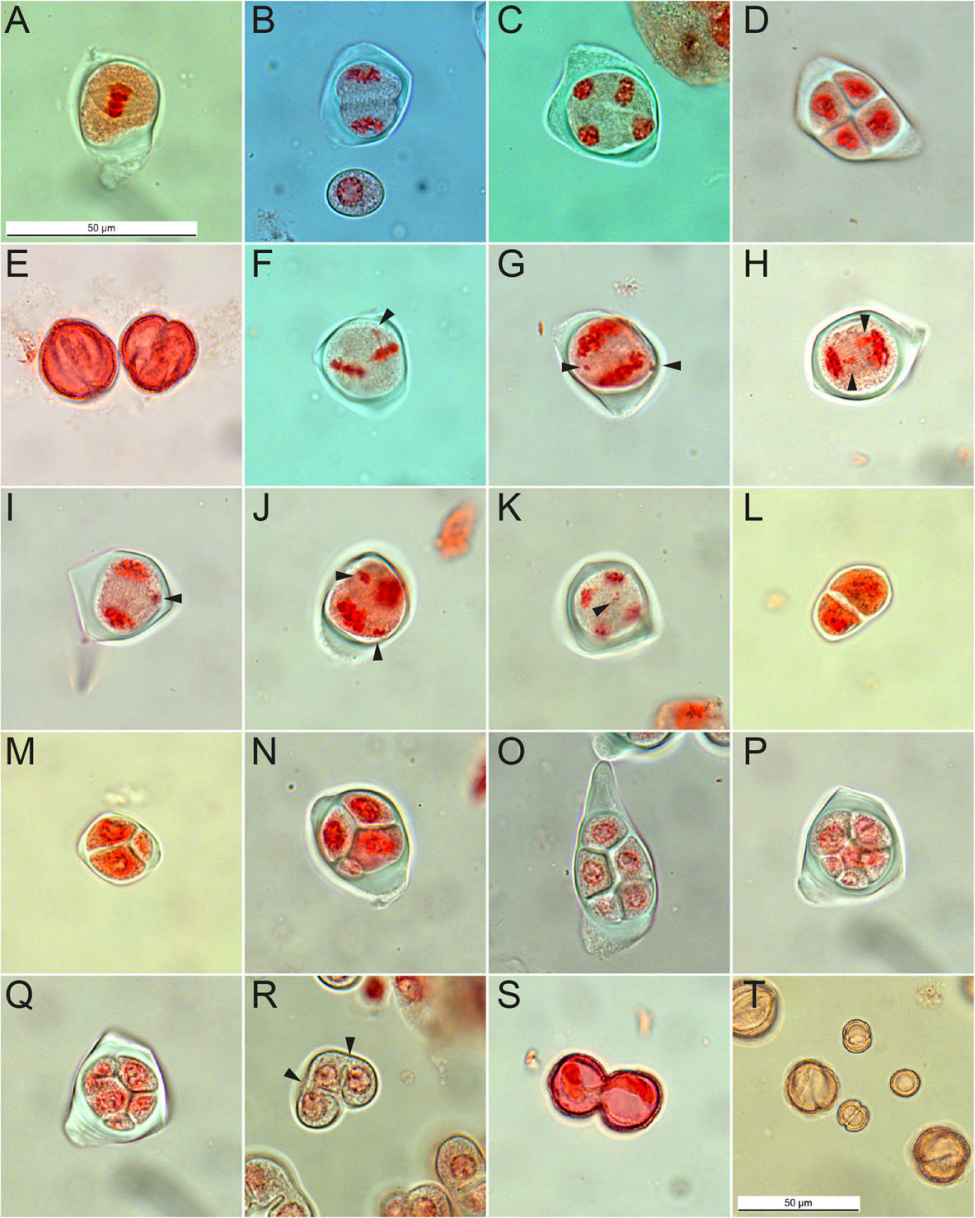
Development of male gametes in *Ranunculus* plants. **a**. – **e**.) Regular meiosis of PMCs, **a**.) PMC at metaphase I, **b**.) PMC at telophase I during cell plate formation, **c**.) PMC at the end of anaphase II, **d**.) Meiotically developed tetrad of microspores, **e**.) Homogeneous-sized pollen grains, **f**. – **p**.) Various cytological failures in *Ranunculus* PMCs, **f**.) PMC at metaphase II showing a sticky out-of-plate chromosome (arrowhead), **g**. + **h**.) PMCs with lagging chromosomes at anaphase I (arrowhead), **i**. + **j**). PMCs with irregular spindle activity (arrowhead), resulting in abnormal chromosome segregation at anaphase II, **k**.) PMC at anaphase II with several lagging chromosomes, **l**.) A Dyad, **m**.) A Triad, **n**.) Tetrad with three normally sized microspores and one miniature microspore, **o**.) Polyad of five uniformly sized microspores, **p**. + **q**.) Figure of the same sporad at different levels. Polyad with seven microspores at different sizes, **r**.) Incompletely separated microspores. Arrowheads point to connections between the three nuclei-containing microspores, **s**.) Dyad pollen grain, **t**.) Heterogeneously-sized micropollen grains. Genotypes: **a**.) F3 * J6 (22); **b**.) J9A; **c.**, **d**., **g**., **j**.) 10136 (15); **e**.) 10137 (08); **f**.) J6 * F7 (14); **h**., **i**., **k**.) G5A; **l**., **m**., **r**., **s**.) F10 * F7 (04); **n**., **t**.) 10136 (08); **o**.) 10136 (02); **p**., **q**.) G16A. Scale bars = 50 μm

**Fig. 2:**
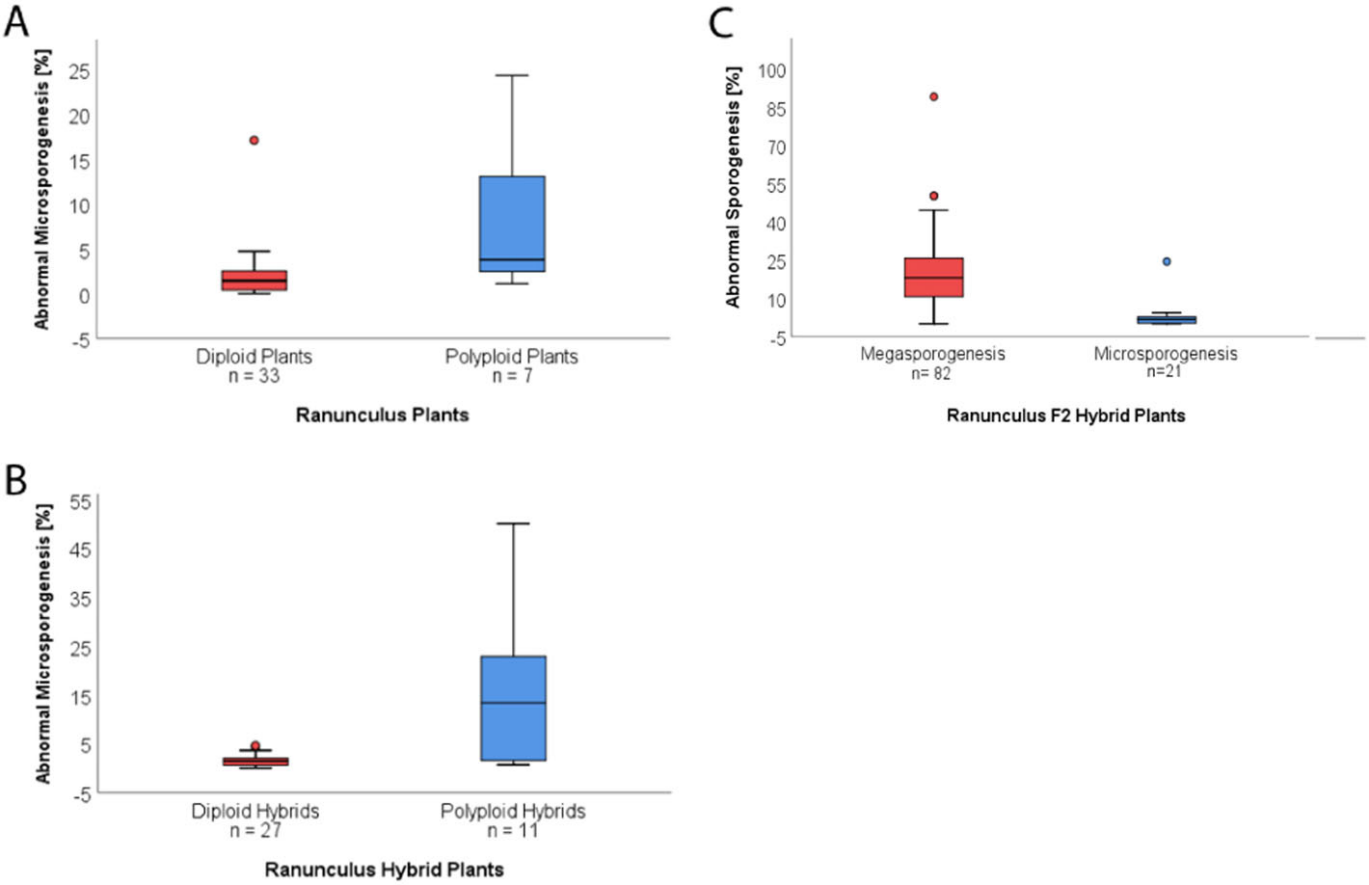
Analysis of irregular male and female sporogenesis in natural and hybrid *Ranunculus* plants. **a**.) Boxplot analysis of percentages of erroneous male meiosis of all three generations. Comparison of diploid and polyploid PMCs revealed a significantly increased frequency of abnormal sporogenesis in polyploid-derived samples (*p* = 0.012, Mann-Whitney-U test). **b**.) Abnormal microsporogenesis depicted for all di- and polyploid hybrid plants, of which allopolyploids showed significantly more irregularities during development than homoploid individuals (*p* = 0.003, Mann-Whitney-U test). **c**.) F_2_ hybrid plants showed different percentages of irregular sporogenesis depending on the sex and ploidy. Statistical comparison of male and female failure in sporogenesis irrespective of ploidy showed a significantly higher frequency of error in female tissue (*p* < 0.001, Mann-Whitney-U test). Outliers are marked as filled circles, the box represents the interquartile range and in the boxplots the median is displayed

In order to determine whether the hybrid character or the ploidy level of *Ranunculus* plants has an influence on the male gametes during meiotic division, more than 10,000 PMCs were analysed for abnormalities (Table 1; Supplementary Data Table S1). The overall frequency of abnormal meiosis in tested male gametes was 5.42%, while the remaining 94.58% resulted in four normal microspores of the same size (Table 1, Fig. 1d). Although, the comparison of abnormal meiotic cell division between the three different plant generations (parents, F_1_ and F_2_ hybrids) did not show significant differences, a significantly higher frequency of faulty microsporogenesis was found in polyploid samples (mean 8.59% ± 9.84 STD, median 3.73%, *p* = 0.012) compared to diploid ones (mean 2.09% ± 3.05 STD, median 1.43%; Table 1, Fig. 2a). In addition, erroneous male gamete formation in all hybrid plants was analysed, including the young, natural hybrid, revealing significantly more failures during sporogenesis in allopolyploid samples (mean 13.28% ± 16.42 STD, median 4.18%, *p* = 0.003) in contrast to homoploid *Ranunculus* individuals (mean 2.11% ± 3.19, median 1.45%; Table 1; Fig. 2b).

**Table 1:** Analysis of male development in di- and polyploid *Ranunculus* gametes during sporogenesis. Mean percentages of normal and abnormal sporogenesis were determined by orcein staining and bright field microscopy

Taxa	Ploidy	Plant ID	n (normal, abnormal)	normal sporogenesis (range)	abnormal sporogenesis (range)
Parent species					
*R. notabilis*	2*x*	10137, 9609	923 (916, 7)	0.99 (0.50–0.99)	0.02 (0.01–0.50)
*R. carpaticola*	2*x*	8483, LH040	369 (365, 4)	0.97 (0.94–0.99)	0.03 (0.01–0.05)
*R. cassubicifolius*	4*x*	LH008	324 (314, 10)	0.97 (0.96–0.98)	0.03 (0.02–0.04)
Synthetic F_1_ Hybrids					
R. carpaticola * R. notabilis	2*x*	J, F	3154 (3123, 31)	0.99 (0.99–1.00)	0.01 (0.00–0.01)
R. cassubicifolius * R. notabilis	3*x*	G	645 (615, 30)	0.89 (0.79–0.99)	0.11 (0.11–0.21)
Synthetic F_2_ Hybrids					
R. car. * R. not. * R. car. * R. not.	2*x*	F x F, F x J, J x F, J x J	3653 (3587, 66)	0.98 (0.95–1.00)	0.03 (0.00–0.17)
R. cas. * R. not. * R. cas. * R. not.	3*x*, 4*x*	G x G	211 (181, 30)	0.86 (0.76–0.95)	0.14 (0.05–0.24)
Natural Hybrids					
R. notabilis * R. variabilis (?)	4*x*	10136	1001 (914, 87)	0.82 (0.50–0.99)	0.18 (0.01–0.50)
Diploid Samples			8099 (7991, 108)	0.98 (0.83–1.00)	0.02 (0.00–0.17)
Polyploid Samples			2181 (2024, 157)	0.87 (0.50–0.99)	0.13 (0.01–0.50)
Total			10,280 (10,015, 265)	94.58%	5.42%

**Fig. 3:**
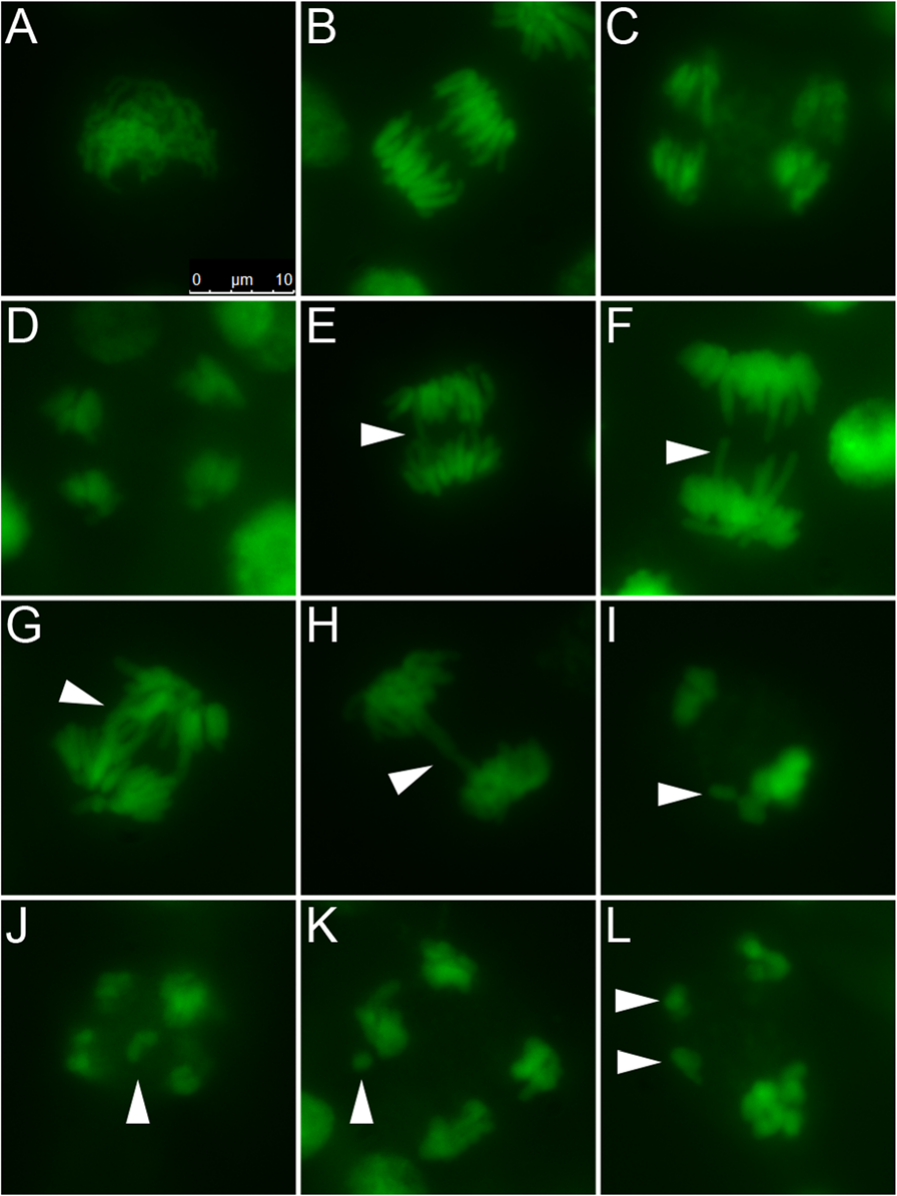
DAPI staining of abnormal chromosome configurations during microsporogenesis of *Ranunculus* plants. **a**. – **d**.) Regular meiosis of PMCs, **a**.) PMC at zygotene, **b**.) PMC at anaphase I, **c**.) PMC at the end of anaphase II, **d**.) PMC at telophase II, **e**. – **l**.) Various developmental failures in *Ranunculus* PMCs, **e**. – **h**.) Sticky chromosomes in PMCs during anaphase I (arrowheads), **i**. – **l**.) PMCs display stickiness due to clumped chromosomes, **i**.) PMC with laggard at anaphase I (arrowhead), **j**. + **k**.) PMCs at anaphase II with lagging chromosomes (arrowheads), **l**.) Erratically separated bivalents at anaphase II (arrowheads). Genotypes: **a**. + **b**., **e**.) F3 * J6 (18); **c**.) J6 * F7 (05); **f**. - **h**.) F3 * J6 (09); **d**., **i**., **l**.) F3 * J6 (30); **j**., **k**.) F3 * J6 (03). Scale bar = 10 μm

Various abnormalities at different meiotic stages were identified in male meiocytes of all *Ranunculus* hybrid generations independently of ploidy levels. Irregularities included lagging chromosomes and chromatin bridges at metaphase II (Fig. 1f). At anaphase I laggards and sticky chromosomes and disoriented spindle activities were detected (Fig. 1g, h, i, Fig. 3e - h). Disoriented spindle activity, as well as scattered chromosomes, occurred during anaphase II (Fig. 1j). In addition, micronuclei were formed during telophase II (Fig. 1k, Fig. 3j - l). The consequence of the described failures during male sporogenesis led to the formation of dyads, triads and polyads, instead of a microspore tetrad (Fig. 1l – q). In turn, incompletely separated and heterogeneous-sized microspores resulted in *Ranunculus* pollen grains of different sizes, of which the micronuclei-derived pollen grains are much smaller than normal pollen (Fig. 1r - t).

### Female sporogenesis and emergence of apospory

Megasporogenesis of three polyploid *Ranunculus* F_2_ hybrid individuals, derived from two different crosses (G1 * G9, G16A * I2A), was analysed for signs of abnormal, aposporic development (Supplementary Data Fig. S1; Table S1). Overall, development of 186 ovules was evaluable because of the small number of formed flower buds by polyploid synthetic F_2_ hybrids and the difficulties to find the developmental stadium of interest. Normal megasporogenesis was detected in 48.92% of the ovules (Table 2; Supplementary Data Table S1). Regular meiotic division was indicated by the presence of a functional megaspore (FM) at the end of the germline, closest to the chalazal pole, while the other three meiotic products were already aborted. Additional to this, apospory was identified in 12.37% of the analysed F_2_ ovules (Table 2). Characteristic for this type of meiosis bypass is the occurrence of an aposporous initial cell (AIC) close to the FM, which is known to dominate ES formation from that point on and results in the abortion of the FM. The remaining 38.71% of the analysed ovules were found to be dead (Table 2). Furthermore, a comparison of di- and polyploid F_2_ hybrid samples for failure during meiotic cell division was done, which resulted in non-significant differences between these two groups (*p* = 0.241, Mann-Whitney-U test).

**Table 2:** Analysis of female development in di- and polyploid *Ranunculus* plants. Mean percentages of normal meiotic cell division, abnormal meiosis and full ovule abortion were investigated by DIC microscopy

Taxa	Ploidy	Plant ID	n	normal meiosis (range)	abnormal meiosis (range)	aborted meiosis (range)
Parent species [32]						
*R. notabilis*	2*x*		86	0.96 (0.94–1.00)	0.00	0.04 (0.00–0.05)
*R. carpaticola*	2*x*		135	0.84 (0.83–0.90)	0.00	0.16 (0.10–0.18)
*R. cassubicifolius*	4*x*		98	0.95 (0.94–0.90)	0.00	0.05 (0.00–0.06)
Synthetic F_1_ Hybrids [32]						
R. carpaticola * R. notabilis	2*x*	J, F	257	0.67 (0.44–1.00)	0.11 (0.00–0.33)	0.22 (0.00–0.56)
R. cassubicifolius * R. notabilis	3*x*	G	191	0.69 (0.54–0.87)	0.15 (0.07–0.32)	0.15 (0.00–0.29)
Synthetic F_2_ Hybrids						
R. car. * R. not. * R. car. * R. not [40].	2*x*	F * F, F * J, J * F, J * J	4811	0.63 (0.45–0.82)	0.16 (0.08–0.26)	0.21 (0.00–0.39)
R. cas. * R. not. * R. cas. * R. not.	3*x*, 4*x*	G * G	186	0.49 (0.06–0.66)	0.12 (0.06–0.15)	0.39 (0.19–0.88)

### Comparison of male and female sporogenesis in synthetic *Ranunculus* F_2_ hybrids

Irregularities were observed in F_2_ hybrids of both, female and male sporogenesis, at different percentages (Fig. 2c, Tables 1, 2; Supplementary Data Table S1). Therefore, the frequencies of abnormal male and female sporogenesis were analysed for differences, revealing a significantly stronger defective meiosis on the female than on the male side (Fig. 2c).

### Generalized linear mixed effect model analysis of sporogenesis in *Ranunculus*

In order to uncover and recess potential connections between the occurrence of deleterious errors in sporogenesis and certain characteristics of the studied plants, GLMM and Chi-squared analyses were performed (Table 3; Supplementary Data Table S1, S2). Polyploid *Ranunculus* plants showed a significantly higher frequency of erroneous microsporogenesis than diploid samples (*p* < 0.001), and a similar negative relation was observed for hybridization. According to this, hybrid plants of the F_2_ generation developed significantly more abnormal male gametes than plants of the non-hybrid parent (*p* < 0.05) and the F_1_ generation (*p* < 0.01). In addition, accumulative effects of *ploidy level* and *generation* were explored by GLMM, indicating weakly but non-significant increased failures of microsporogenesis in diploid *Ranunculus* F_2_ hybrids compared to both, polyploid parent plants (*p* = 0.08) and polyploid F_1_ hybrids (*p* = 0.09). The impact of polyploidy on developmental behaviour was additionally investigated in female sporogenesis of F_2_ hybrids, inferring no significant differences (*p* = 0.46). Furthermore, the total F_2_ dataset, comprising mega- and microsporogenesis measurements, was consecutively tested for an influence of *ploidy level* and *sex* on gamete formation. A highly significant relation between errors during female sporogenesis and plant polyploidy was observed (*p* < 0.001) as well as between faulty microsporogenesis in diploid F_2_ hybrids and megasporogenesis in polyploid F_2_ plants (*p* < 0.001).

**Table 3:** Generalized mixed-effect model (GLMM) analyses discovering manipulating effects influencing the error rate of male and female meiosis and sporogenesis in *Ranunculus* with regard to *ploidy level*, *generation* and *sex*. Calculations were based on 115 *Ranunculus* plants and more than 13,000 individual data points. Statistical computation procedure in R is depicted. Regression estimate and *p* value are calculated by GLMM analysis as the tested factor is referred to the test and base line categories. *p < 0.05, **p < 0.01, ***p < 0.001 for statistical significance of the test. For more detailed statistical info see Supplementary Data Table S2

Subset	n	Tested factor(s)	Base line categories	Test categories	GLMM Regression Estimate	*p* value
Male	9193	*ploidy level*	2*x*	4*x*	2.19	***
		*generation*	F_2_	P	- 0.77	*
				F_1_	- 0.63	**
		combined effect	2*x*, F_2_	4*x*, P	- 0.88	0.08
				4*x*, F_1_	- 0.60	0.09
Female	3660	*ploidy level*	2*x*	4*x*	0.17	0.46
Male/ Female	7438	*ploidy level*	2*x*	4*x*	0.17	0.46
		*sex*	female	male	- 2.44	***
		combined effect	2*x*, female	4*x*, male	2.02	***

Chi-squared tests were done to support GLMM analyses, obtaining corroborative results (Supplementary Data Fig. S2, Table S2). Highly significant differences in microsporogenesis performance were detected between di- and polyploid *Ranunculus* plants of the parental (Χ^2^ = 119.78, df = 1, *p* < 0.001), the F_1_ hybrid (Χ^2^ = 8.42, df = 1; *p* = 0.01), and the F_2_ hybrid (Χ^2^ = 43.32, df = 1, *p* < 0.001) generation (Supplementary Data Fig. S2b). In addition, similar significant differences in error frequency were observed between male and female sporogenesis of F_2_ hybrids (Χ^2^ = 470.82, df = 1, *p* < 0.001; Supplementary Data Fig. S2c).

## Discussion

Hybridization and polyploidization are known to have substantial effects on male and female reproductive programs in angiosperms [11]. Although hybridization was recently shown to play an important role in the onset of apospory in diploid *Ranunculus* plants, its interaction with meiotic behaviour remained unclear [32, 40]. The investigation of chromosomal behaviour at meiosis plus male and female sporogenesis in *Ranunculus* allows first insights into the role of meiosis and sporogenesis for occurrence of apomictic reproduction in hybrid and polyploid plants.

In this study, microsporogenesis progression in di- and polyploid *Ranunculus* plants of natural and hybrid origin were analysed to identify deviations during reproduction that mediate abnormal cytological products. Through a combined analysis of acetic-orcein and DAPI staining, irregularities in polyploid flower buds were identified as significantly higher as in diploid plant tissue. This is striking as the great majority of diploid plants studied here were F_1_ and F_2_ hybrids, which did not differ significantly from their parental diploid species, in regard to frequency of erroneous male sporogenesis (Table 1). Limited viability and fertility of young hybrids are extensively described and therefore, poor hybrid fitness is often taken for granted in case of natural hybrid progeny [12, 15], whereas F_2_ hybrid performance is often worse than the situation in F_1_ progeny but hybrids are not invariably less fit than their parents [9, 44]. Investigations on the influence of polyploidy, in *Ranunculus* hybrid plants only (including the natural allopolyploids), revealed a significantly increased frequency of disturbed microsporogenesis in polyploid versus diploid hybrids (Table 3, Fig. 2b).

Overall, 5.42% of all analysed samples showed an altered course of male sporogenesis (Table 1) with manifold error types, of which problems in bivalent and spindle formation and orientation are thought to be the most dramatic ones. In consequence, these meiotic failures led to abnormally shaped microspores (Fig. 1l - t). A significantly greater proportion of irregularly developed sporads was observed in polyploid *Ranunculus* plants (mean 8.59%, *p* = 0.012), which led to the conclusion that polyploidization in combination with hybridization favours malfunctions in male reproductive development rather than hybridization alone (Fig. 2a). The natural plants under investigation have the same karyotypes [29, 45], and the here included hybrids did not show apparent deviations from this shared karyotype. Hence, meiotic disturbances cannot be explained by the pairing of structurally different chromosome sets. An overview of karyotypes and hybrid formation in the genus *Ranunculus* supports the hypothesis that uniform karyotypes facilitate hybridization events [45] and might lead to less detrimental effects on fitness in newly formed homoploid *Ranunculus* hybrids. The production of dyads, triads and polyads seems to be due to various problems during microsporogenesis. Since meiosis is described to be very sensitive to unbalanced chromosome segregation, either chromosome mispairing likely led to the formation of uni- and multivalents or erroneous spindle activities resulted in unusual gamete generation and pollen [10, 46]. This assumption is supported by the observation of anaphases with an odd number of spindle poles (Fig. 1i). Nevertheless, chromosome mispairing cannot be ruled out because unbalanced chromosome segregation was regularly detected as well (Fig. 1j). In rare cases, plants showed incomplete cell plate assembly, forming unseparated aggregations of poly-nucleated microspores and in consequence, dyad pollen grains (Fig. 1r - s). Sporads, equipped with more than the normal quantity of four meiotic products, were believed to originate from unsuccessful chromosome division that again could be associated with defective spindle function. The detection of dwarf-microspores could be correlated to their genomic content, since in *Arabidopsis* and other model plants pollen size is positively connected to their DNA content [47]. However, this link to genome size was not yet demonstrated in *R. auricomus*, but microscopic pollen studies revealed dwarf and malformed pollen in apomictic taxa [29]. Quantitative pollen analyses in apomictic *Ranunculus kuepferi* found a great variation in pollen size, and dwarf pollen in tetraploids to be inviable [48]. The observed abnormalities during male meiocyte development seem to be relatively common phenomena in polyploid Ranunculaceae. Kumar et al. [49] characterized meiotic progression in tetraploid *Ranunculus* species, collected at the Himalayas. Consistent with the present data, they found several severe meiotic problems including chromosome stickiness, laggards as well as disoriented bivalents. For example, the disoriented chromosome in Fig. 1g may be the result of mispairing plus subsequent missegregation. To estimate whether the obtained results are the consequences of synthetically generated polyploid *Ranunculus* hybrids, additionally, a tetraploid (*R. cassubicifolius*, parent species) and a potential young, natural allopolyploid (Table 4; Supplementary Data Table S1) were included. It is assumed that the latter plants represent natural crosses between *R. notabilis* and *R. variabilis* due to phenotypical reasons as well as due to the fact that a *R. variabilis* population occurs nearby [50]. The frequency of abnormal microsporogenesis was found to be consistent with data of the *Ranunculus* hybrids made by hand-pollination (F_1_; F_2_; Table 4; Supplementary Data Table S1, S2). This finding shows that irregularities can be triggered by hybridization events but can get significantly stronger, when it is combined with polyploidization as well (Table 4, Fig. 2a, b).

**Table 4:** Natural plants and synthetic hybrids of the *Ranunculus auricomus* complex analysed in this study

Generation	*Ranunculus* Plants	Reproduction Mode	Plant ID	Ploidy	Reference
Parent Plants	*R. carpaticola*	Sexual	8483, LH040	2*x*	[32] Supplementary Data Table S3, Supplementary Data Fig. S3
	*R. notabilis*	Sexual	10137, 9609	2*x*	[50]
	*R. cassubicifolius*	Sexual	LH008, LH009	4*x*	Supplementary Data Table S3, Supplementary Data Fig. S3
F_1_ Hybrids	*R. carp.* * *R. not.*	Sexual	F, J	2*x*	[32]
	*R. cassu.* * *R. not.*	Facultative apomictic	G	3*x*	
F_2_ Hybrids	*R. carp.* * *R. not.* * *R. carp.* * *R. not.*	Facultative apomictic	F * F, F * J, J * F, J * J	2*x*	[40]
	*R. cassu.* * *R. not.* * *R. cassu.* * *R. not.*	Facultative apomictic	G * G	3*x*, 4*x*	
Natural Hybrids	R. not. * R. variabilis (?)	unknown	10136	4*x*	[50]

Furthermore, the age and degree of diploidization seem to play a crucial role for meiosis function because the tetraploid *R. cassubicifolius*, which is at least 80,000 years old [35], displayed very low frequencies of abnormal male gamete formation that are similar to that of diploid *Ranunculus* material (Table 4). Polyploidy is common in angiosperms and these plants are regarded as evolutionary fit, which might be due to a long diploidization process that is stabilizing meiocyte formation and genetic/epigenetic regulatory mechanisms [10, 19]. Thus, *R. cassubicifolius* plants are assumed to have overcome the bottleneck of currently polyploidized plants like in our natural hybrid samples [10]. The analysis of sporogenesis in male organs of F_1_ *Ranunculus* hybrids has shown an increase in errors comparing di- and polyploid samples, which is consistent with the rest of this study but in contrast to the data gathered by Hojsgaard et al. [32]. There, microsporogenesis was described as “regularly and normally proceeding”. These discrepancies could be explained by the smaller sample size of the previous study.

Results raise the old question of whether polyploidization, hybridization or a combination of both is the reason for the switch and fixation of apomixis. The two evolutionary forces are commonly held responsible for the emergence of apomictic seed formation in plants either alone or in combination. Researchers intensively debate about this topic, since both factors do occur much more frequently uncoupled from apomixis as well [20, 23]. In neopolyploids, apomixis might occur as a short-term transitional stage resulting in unreduced gamete formation, but then continued via a reversal to obligate sexuality in established polyploids [23, 51]. For such instable, occasional shifts to apomixis, effects of different environmental stress factors on mode of reproduction might also play a role [43, 52]. Other potential reasons for emergence of apomixis may be certain genetic and epigenetic dislocations in angiosperm genomes provoked by hybridization or allopolyploidization, respectively [10, 51, 53]. This hypothesis is supported by several studies that observed heterochronic alterations in female development of synthetic *R. auricomus* hybrids [32, 40], which could be due to reversible epigenetic silencing [54]. Nonetheless, such alterations could be also a consequence of previous karyotypic changes after chromosome loss, rearrangements or missegregation. More substantial proofs are required to test these hypotheses, and they are not mutually exclusive.

In order to draw an elusive picture of meiotic progression in aposporous hybrid *Ranunculus* samples, female sporogenesis was compared to male data. Female sporo- and gametogenesis in the parental plant and F_1_ generation were analysed previously by Hojsgaard et al. [32] and the situation in F_2_ *Ranunculus* plants by Barke et al. [40] and Ulum et al. [43]. These experiments have exclusively shown sexual ES formation for parental individuals, while in F_1_ and F_2_ hybrids apospory was detected [[32, 40]; Table 2]. The formation of an AIC and the abortion of meiotic products are well known, characteristic features of gametophytic apomixis [e.g. [20, 55]]. Since aposporous initials always appear at the end of megasporogenesis, but neither independently nor during the course of meiosis, it is likely that the final meiosis failure has an effect on AIC formation [26, 27, 32, 40, 43], while the AICs have no more influence on previous meiosis progression. It is therefore probably less relevant whether meiosis is disturbed at an earlier or later stage, as only the end-product of meiosis correlates with appearance of aposporous initials. The fourth megaspore, close to the chalazal pole, is the only remaining cell of the megaspore tetrad, and is conventionally still called “functional megaspore” (FM), although it is doubtful whether this cell is functional due to manifold meiotic errors. It aborts sooner or later during embryo sac formation. Aposporous cells are located adjacent to the megaspore tetrad, establishing direct contact with the FM. Therefore, another intensively discussed possibility is cell-to-cell crosstalk that could trigger the abortion of the sexually derived cells and/or the formation of the aposporous one [23, 55–57]. In this study, recently collected data for megasporogenesis of polyploid *Ranunculus* F_2_ plants were amended with results of synthetic diploid F_2_ hybrids [40]. This analysis revealed similar frequencies for occurrence of apospory in di- and polyploid ovaries (Table 2). However, an overall comparison of female and male sporogenesis resulted in a significantly higher error rate in female organs rather than on the male side (Fig. 2c). Monosporous development in *Ranunculus* increases the risk of negative consequences of meiotic errors, as always just one of the megaspores is left to continue ES establishment. If this megaspore (the chalazal one) has revealed an incomplete chromosome set, e.g. due to irregular chromosomal segregation, it cannot be replaced by the other megaspores of the tetrad. No tendencies towards polysporic embryo sac development were observed, as reported for other apomictic plants [58]. By contrast, male sporogenesis in *Ranunculus* leads to four haploid microspores, each continuing microgametogenesis within one pollen grain. Therefore, reduced male fertility, accomplished by abnormal meiotic behaviour and disturbed microsporogenesis and -gametogenesis, has not such serious quantitative consequences as in ovaries. The remaining intact pollen grains with functional gametes are numerous enough for successful fertilizations. Pseudogamous apomicts like *R. auricomus* plants need pollen for fertilization of polar nuclei for proper endosperm formation. Hence, selection will favour the maintenance of a male function even in apomictic plants [59]. In contrast, ovules are much less numerous, the pollen-ovule ratio ranges in *R. auricomus* from 652 to 1684 [42]. Unlike the situation in pollen, the death of the functional megaspore (whole germline) easily jeopardizes the female reproduction success of the plant. Thus, selection pressure for an alternative apomeiotic developmental pathway is acting much harder on female than on male function in a hermaphroditic plant. In this study, less than 50% of megasporogenesis in polyploid plants followed the sexual reproduction pathway, while nearly 40% of analysed ovules showed abortion and approx. 10% formation of an aposporous initial (Table 2). Sexual ES formation in diploid hybrid samples made up more than 60%, 20% of the germlines were fully aborted and 16% developed aposporously [Table 2, [40]]. Thus, the onset of apomixis, as already Darlington [22] proposed, really seems to be an escape from hybrid sterility, but only on the female side. Nonetheless, seed formation in Barke et al. [40] was only analysed in diploid plants due to mentioned high seed abortion rates. The effective influence of combined hybridization and polyploidization in *Ranunculus* was mainly observed on embryo sac formation.

Diploid hybridization appears to be a less effective trigger for apomixis than allopolyploidy. This hypothesis is in line with the general scarcity of diploid hybrids expressing apomixis in natural systems [23]. The most prominent exception is found in the genus *Boechera*, where apomixis is fully functional in diploid hybrids [60]. But, in this genus dramatic chromosomal rearrangements were observed in diploid apomicts [60], and the apomictic diploid hybrid lineages originated from combinations of strongly disparate genomes [61]. Otherwise, apomictic seed formation in natural diploid populations appears in very low frequencies [reviewed by [23]] and could be also due to environmentally induced disturbance of sexual development [52]. To which extent female meiotic irregularities in diploid hybrids are responsible for the establishment of apomixis, however, needs to be studied further. Our study showed a significantly higher frequency of microsporogenesis errors in polyploid hybrids than in homoploid ones (Fig. 2b; Table 1), but no differences of ploidy levels in the success of megasporogenesis.

## Conclusion

This study sheds new light on cytological processes that happen in young allopolyploids and diploid *Ranunculus* hybrids and their role in apomictic reproduction. Results suggest that polyploidization has a much stronger detrimental effect on male meiosis than homoploid hybridization. Irregularities during sporogenesis are much more frequent in female than in male development, even in the same plant. The correlation of failure of megasporogenesis to the appearance of apospory suggests indeed that disturbed megasporogenesis could be a functional trigger for apomixis, but this appears to be ploidy-dependent. It was concluded that differential selective pressures act on male and female meiosis: While female development is constrained to circumvent meiosis to produce any functional embryo sac, male development can continue with a disturbed meiotic pathway, with selection acting on the huge mass of pollen that is still produced.

## Methods

### Plant material

In this study, three generations of wild and hybrid plants were used. The parent plants were natural, diploid allogamous *R. carpaticola* and *R. notabilis*; and natural, tetraploid, allogamous *R. cassubicifolius* that all have been collected from wild populations (Tables 4, 5, S1) and were determined to reproduce sexually [32]. Homo- and heteroploid hybrid plants had been generated by manual crossings in 2006, which resulted in diploid F_1_ hybrids (F, J plants; Table 4; Supplementary Data Table S1) obtained from *R. carpaticola* * *R. notabilis* crosses and triploid F_1_ individuals (G plants; Table 4; Supplementary Data Table S1) gained by crossing *R. cassubicifolius* * *R. notabilis* [32]. Additionally, between 2010 and 2012, a second hybrid generation was produced using F_1_ plants that have shown apospory [32]. F_2_ individuals with F and/or J parents were found to be diploid and aposporous [[40], Table 4; Supplementary Data Table S1], while hybrids descending from G parents were determined to be tri- and tetraploid [[40], Table 4; Supplementary Data Table S1]. Since the original parental plants were no longer alive, we collected individuals from the same populations between 2011 and 2018 for the study here. In addition, tetraploid *R. notabilis* hybrid plants from another population that was previously described as diploid [[50], Table 4; Supplementary Data Table S1]. We regard these plants as recently formed backcrosses with pollen from 4*x R. variabilis*, a species, which occurs at the same location [50]. All analysed plants in this study are grown outdoors in the old botanical garden of the Albrecht-von-Haller Institute for Plant Sciences at the University of Goettingen, Germany under the same climatic conditions.

**Table 5:** List of wild collected natural *Ranunculus* plants analysed in this study incl. Herbarium voucher depositories - GOET (Herbarium University Goettingen) and WU (Herbarium University of Vienna). No permits were required for the collection of these *Ranunculus* samples

*Ranunculus* Plants	Plant ID	Localities (Collector, Date)	Plant Identification (Herbarium)
*R. notabilis*	9609, 10137	Austria, Burgenland, Strem valley, Moschendorfer forest (Hörandl, 8 May 2011)	Hörandl (WU)
R. not. * R. var. (?)	10136	Austria, Burgenland, Strem valley, Moschendorfer forest (Hörandl, 8 May 2011)	Hörandl (WU)
*R. carpaticola*	8483	Slovakia, Slovenské rudohorie, Revúca, hill Skalka (Hörandl, 5 May 1998)	Hörandl (WU)
*R. carpaticola*	LH040	Slovakia, Slovenské rudohorie, Banskobystrický kraj (Hodač, 3 May 2018)	Hörandl (GOET)
*R. cassubicifolius*	LH008	Austria, Lower Austria, Ybbs valley, Eisenwurzen (Hodač, 1 May 2017)	Hörandl (GOET)
*R. cassubicifolius*	LH009	Austria, Lower Austria, Ybbs valley, Eisenwurzen (Hodač, 1 May 2017)	Hörandl (GOET)

### Determination of ploidy and mode of reproduction

Ploidy and mode of reproduction of the hybrids are documented in Hojsgaard et al. [32] for the F_1_ and in Barke et al. [40] for the F_2_ generation. The newly collected individuals of the parental species were checked for ploidy and mode of reproduction by flow cytometry following protocols of Barke et al. [40]. Flow cytometric seed screening confirmed sexual reproduction for the *R. notabilis* and *R. cassubicifolius* individuals (Supplementary Data Table S3; Supplementary Data Fig. S3).

### Flower bud fixations

For studying male meiosis in natural and artificial hybrid *Ranunculus* plants, small flower buds with a maximal diameter of 5 mm were harvested in spring and were directly fixed in ethanol: acetic acid (3: 1) and stored until usage at 4 °C. Flower buds fixed with this method were used for orecin staining and chromosome spreads.

For the analysis of megasporogenesis, flower buds of a minimal diameter of 5 mm were collected and fixed in FAA solution (formaldehyde: acetic acid: ethanol: dH_2_O; 2: 1: 10: 3.5). After an incubation period of 48 h at room temperature the fixative solution was carefully exchanged by 70% ethanol and stored at room temperature until analysis of female development [40].

### Pollen mother cell orcein staining

Male sporogenesis was analysed by dissecting stamina from fixed flower buds on a microscopic slide, while adding a droplet of 2% (w/v) lactopropionic orcein solution to the plant tissue. After installing the cover slip, mild thumb pressure was applied to the sample in order to release and stain the pollen mother cells (PMCs).

### Chromosome spreads

The behaviour of chromosomes during male meiosis was investigated using the widely known chromosome spreading technique [47, 62] with several minor modifications. Fixed flower buds were washed twice in ddH_2_O and once in citrate buffer (pH 4.8) until no “clouds” of fixative were detected. Plant tissue digestion was accomplished by incubation of the buds in an enzyme mixture made from 5% (w/v) pectinase (Sigma-Aldrich Chemie GmbH, Taufkirchen, Germany) and 5% cellulase (Onozuka R10; SERVA Electrophoresis GmbH, Heidelberg, Germany) in citrate buffer at 37 °C in a moisture chamber for 5 h. After digestion enzyme mixture was carefully exchanged by citrate buffer and samples were stored for 1 h at 4 °C. A single flower bud was transferred to a microscopic slide, containing one droplet of 60% acetic acid, in which the plant tissue was squashed using a bent dissecting needle. Subsequently, the microscopic slide was heated on a hotplate at 45 °C and plant tissue was uniformly spread across the warm slide. Therefore, the sample was submerged with freshly made, ice-cold ethanol: acetic acid (3: 1) fixative and then air-dried. Chromosome staining was achieved by adding 20 μl DAPI (1 μl/ ml; 4′,6-diamidino-2-phenylindole; Carl Roth GmbH + Co. KG, Karlsruhe, Germany) in VECTASHIELD^®^ antifade mounting medium (VECTOR LABORATORIES, INC., Burlingame, CA, USA) and a cover slip to the sample. Finally, the sample was incubated overnight in the dark at 4 °C to develop fully stained chromosomes.

### Female development

The megasporogenesis study of polyploid *Ranunculus* F_2_ hybrids was performed using the well-documented differential interference contrast (DIC) microscopy [32, 40]. Prefixed flower buds were dehydrated by incubation for 30 min in 95 and 100% ethanol. In a subsequent treatment with an increasing dilution series of methyl salicylate (25; 50; 85; 100%; Carl Roth GmbH + Co. KG, Karlsruhe, Germany) in ethanol the flower bud tissue was cleared [40, 63]. For microscopy entire *Ranunculus* ovaries were dissected from the cleared plant tissue and mounted on a microscopic slide in a droplet of pure methyl salicylate.

### Microscopy

Visualization of male meiotic chromosomes and of female sporogenesis in *Ranunculus* samples was carried out with a Leica microscope DM5500B. Images of the orcein-stained PMCs and the cleared ovaries were taken with a DFC 450C camera and LAS V41 software (Leica Microsystems, Wetzlar, Germany). For fluorescent picture imaging, the same microscope equipped with the DFC 365FX camera, the FLUO-filter cube A4 and the LAS AF 3.1.0 software was applied (Leica Microsystems CMS GmbH, Wetzlar, Germany).

### Statistical analyses

Percentages of abnormal male and female meiosis and sporogenesis were calculated for each individual, and shown with boxplots. Descriptive statistical analyses and tests for significant differences of two groups (either diploid versus polyploid PMCs /ovules or female versus male meiosis) were done by applying a Mann-Whitney-U test, due to not normally distributed data, using IBM SPSS Statistics 24 (IBM Deutschland GmbH, Ehningen, Germany).

To investigate the influence of sex, ploidy level and generation on sporogenesis, generalized linear mixed effect model (GLMM) analyses were performed using R package *lme4* v1.1–20 [64]. Sporogenesis was defined as response variable and determined as a binominal state; either normal (0) or abnormal (1). Binominal character distribution in the response variable enabled the application of GLMM analyses from the binomial error structure family [65]. The explanatory variables *sex*, *ploidy level* and *generation* were defined as categorical, occupying exactly one of a set of non-overlapping options; *sex*: male or female, *ploidy level*: diploid or polyploid, *generation*: P, F_1_ or F_2_. Interactions were allowed between explanatory variables within each GLMM analysis. The three sampled *Ranunculus* species (Table 4) were defined as random factor within GLMM analyses to control for interspecific effects. The data for natural allopolyploid *R. notabilis* * *R. variabilis* specimens was excluded from further analyses to prevent introduction of a potential bias regarding origin of polyploidy.

In order to test whether *ploidy level*, *generation* or both influence the course of microsporogenesis, GLMM analyses were executed for each categorical factor and in F_2_ *Ranunculus* material, the impact of *ploidy level* on female sporogenesis was analysed. To infer whether male and female sporogenesis were differently affected in F_2_ hybrids, the data of Barke et al. [40] on embryo sac development were combined with those herein to produce a total F_2_ dataset. In addition, verification of adverse effects on sporogenesis, caused by *ploidy levels*, *sex* or a combination of both, was done by consecutive GLMM analyses of the total F_2_ dataset. A Laplace approximation was employed to fit the GLMMs to the data using the R function *glmer()* [64]. Chi-squared tests were performed to test for different effects of explanatory variables. Results of GLMM analysis and Chi-squared tests were plotted to bar graphs using R v3.5.2 (R Foundation for Statistical Computing 2018).

## Supplementary information

**Additional file 1:** **Table S1:** Number of cytological abnormalities detected in micro- and megasporogenesis in *Ranunculus*. **Table S2:** More detailed information on the generalized mixed-effect model (GLMM) analyses. These analyses observed effects changing the error frequency of micro- and megasporogenesis in *Ranunculus* with regard to ploidy, generation and sex. Calculations were based on 115 *Ranunculus* plants and more than 13,000 individual data points. R calculation output is visualized including standard error and z value. Regression estimate and p value are calculated by GLMM analysis and the tested factor is referred to the test and base line categories. **Table S3:** Mean peak indices of reproductive mode of different *Ranunculus* populations. **Fig. S1:** Asexual ES formation in an ovule of a diploid *Ranunculus* F_2_ hybrid (taken from [40]). **Fig. S2:** Chi-squared analyses of erroneous mega- and microsporogenesis in natural and hybrid *Ranunculus* plants. **Fig. S3:** Representative flow cytometry histograms of *Ranunculus* seeds.

## Data Availability

All data generated or analyzed during this study are included in this published article [and its supplementary information files]. Additional microscopic images (original resolution) are available from the corresponding author on reasonable request.

## Abbreviations

AIC: Aposporous initial cell
DIC: Differential interference contrast
ES: Embryo sac
FM: Functional megaspore
GLMM: Generalized linear mixed effect model
PI: Peak index
PMC: Pollen mother cell
STD: Standard deviation
