# Supplementary material for: The relation of meiotic behaviour to hybridity, polyploidy and apomixis in the Ranunculus auricomus complex (Ranunculaceae)
Source: BMC Plant Biol. 2020 Nov 17;20:523. doi: 10.1186/s12870-020-02654-3 (PMC7672892; doi:10.1186/s12870-020-02654-3)
Supplement: Supplementary file 1 — Additional file 1: Table S1: Number of cytological abnormalities detected in micro- and megasporogenesis in Ranunculus. Table S2: More detailed information on the generalized mixed-effect model (GLMM) analyses. These analyses observed effects changing the error frequency of micro- and megasporogenesis in Ranunculus with regard to ploidy, generation and sex. Calculations were based on 115 Ranunculus plants and more than 13,000 individual data points. R calculation output is visualized including standard error and z value. Regression estimate and p value are calculated by GLMM analysis and the tested factor is referred to the test and base line categories. Table S3: Mean peak indices of reproductive mode of different Ranunculus populations. Fig. S1: Asexual ES formation in an ovule of a diploid Ranunculus F2 hybrid (taken from [40]). Fig. S2: Chi-squared analyses of erroneous mega- and microsporogenesis in natural and hybrid Ranunculus plants. Fig. S3: Representative flow cytometry histograms of Ranunculus seeds. [file 12870_2020_2654_MOESM1_ESM.zip › Suppl. Fig. S1_former Suppl. Fig. S3.docx]

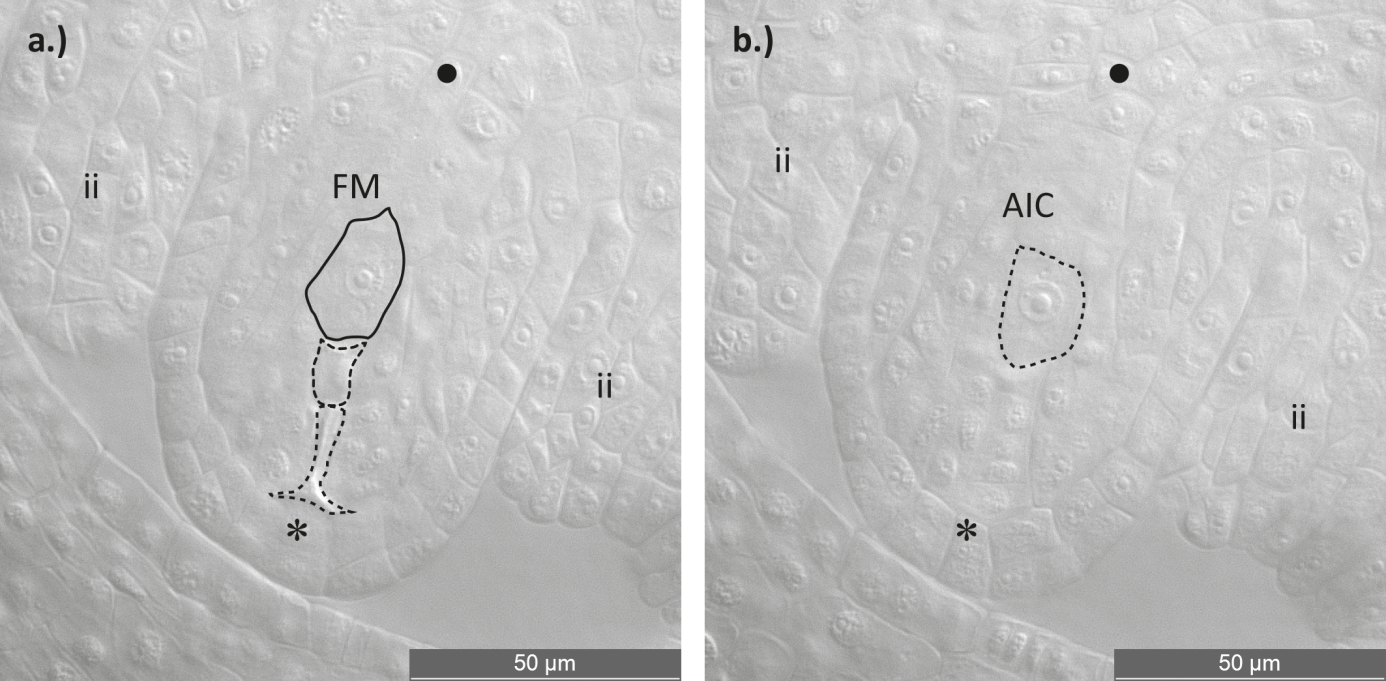


Figure S1: Asexual embryo sac formation in an ovule of a diploid *Ranunculus* F_2_ hybrid [40]. a.) Ovule developing a functional mega­spore. The germ line shows the four meiotic products, of which three megaspores are already aborted. Exclusively, the one closed to the chalazal pole survived and developed into a functional megaspore. b.) Depicted is the identical ovule as in Fig. a. but displaying one cell layer above the germ line, showing an aposporous initial cell. Plant individual: J10 x J30 (12). FM, functional mega­spore; AIC, aposporous initial cell; ii, inner integuments; *, micropylar pole; ●, chalazal pole. Scale bar: 50 µm

.
