# Supplementary material for: The relation of meiotic behaviour to hybridity, polyploidy and apomixis in the Ranunculus auricomus complex (Ranunculaceae)
Source: BMC Plant Biol. 2020 Nov 17;20:523. doi: 10.1186/s12870-020-02654-3 (PMC7672892; doi:10.1186/s12870-020-02654-3)
Supplement: Supplementary file 1 — Additional file 1: Table S1: Number of cytological abnormalities detected in micro- and megasporogenesis in Ranunculus. Table S2: More detailed information on the generalized mixed-effect model (GLMM) analyses. These analyses observed effects changing the error frequency of micro- and megasporogenesis in Ranunculus with regard to ploidy, generation and sex. Calculations were based on 115 Ranunculus plants and more than 13,000 individual data points. R calculation output is visualized including standard error and z value. Regression estimate and p value are calculated by GLMM analysis and the tested factor is referred to the test and base line categories. Table S3: Mean peak indices of reproductive mode of different Ranunculus populations. Fig. S1: Asexual ES formation in an ovule of a diploid Ranunculus F2 hybrid (taken from [40]). Fig. S2: Chi-squared analyses of erroneous mega- and microsporogenesis in natural and hybrid Ranunculus plants. Fig. S3: Representative flow cytometry histograms of Ranunculus seeds. [file 12870_2020_2654_MOESM1_ESM.zip › Suppl. Fig. S2.doc]

Figure S1: Chi-squared analyses of erroneous mega- and microsporogenesis incl. meiotic cell division in natural and hybrid Ranunculus plants. a.) Bar graph depicting percentages of sporogenetic failures in R. cassubicifolius, polyploid F_1_ and F_2_ hybrids as well as in natural polyploid hybrids, showing no significant difference (X^2^ = 1.57, df = 1, p = 0.21). b.) Highly significant differences between di- and polyploid parent plants (X^2^ = 119.87, df = 1, p < 0.001), F_1_ hybrids (X^2^ = 8.42. df = 1, p = 0.01) and F_2_ hybrids were found (X^2^ = 43.32, df = 1, p < 0.001). c.) Comparison of megasporogenesis in di- and polyploid F_2_ hybrids revealed no significant difference (X^2^ = 0.37, df = 1, p = 0.54), whereas highly significantly more errors in microsporogenesis were found in polyploid F_2_ plants compared to diploid F_2_ individuals (X^2^ = 119.87, df = 1, p < 0.001). Overall, significantly more abnormal sporogenesis was observed in female than in male gamete development (X^2^ = 470.82, df = 1, p < 0.001).
