# Supplementary material for: The relation of meiotic behaviour to hybridity, polyploidy and apomixis in the Ranunculus auricomus complex (Ranunculaceae)
Source: BMC Plant Biol. 2020 Nov 17;20:523. doi: 10.1186/s12870-020-02654-3 (PMC7672892; doi:10.1186/s12870-020-02654-3)
Supplement: Supplementary file 1 — Additional file 1: Table S1: Number of cytological abnormalities detected in micro- and megasporogenesis in Ranunculus. Table S2: More detailed information on the generalized mixed-effect model (GLMM) analyses. These analyses observed effects changing the error frequency of micro- and megasporogenesis in Ranunculus with regard to ploidy, generation and sex. Calculations were based on 115 Ranunculus plants and more than 13,000 individual data points. R calculation output is visualized including standard error and z value. Regression estimate and p value are calculated by GLMM analysis and the tested factor is referred to the test and base line categories. Table S3: Mean peak indices of reproductive mode of different Ranunculus populations. Fig. S1: Asexual ES formation in an ovule of a diploid Ranunculus F2 hybrid (taken from [40]). Fig. S2: Chi-squared analyses of erroneous mega- and microsporogenesis in natural and hybrid Ranunculus plants. Fig. S3: Representative flow cytometry histograms of Ranunculus seeds. [file 12870_2020_2654_MOESM1_ESM.zip › Suppl. Fig. S3_former Suppl. Fig. S1.doc]

Figure S3: Representative flow cytometry histograms of Ranunculus seeds (a.–c.). General peak labeling: 1 embryo peak and 2 endosperm peak. a.) R. cassubicifolius sexual seed with a tetraploid embryo and a hexaploid endosperm. b.) R. carpaticola sexual seed with a diploid embryo and a triploid endosperm. c.) R. cassubicifolius sexual seed with a tetraploid embryo and a hexaploid endosperm. Populations: a.) LH008, b.) LH040, c.) LH009.
