# Supplementary material for: The relation of meiotic behaviour to hybridity, polyploidy and apomixis in the Ranunculus auricomus complex (Ranunculaceae)
Source: BMC Plant Biol. 2020 Nov 17;20:523. doi: 10.1186/s12870-020-02654-3 (PMC7672892; doi:10.1186/s12870-020-02654-3)
Supplement: Supplementary file 1 — Additional file 1: Table S1: Number of cytological abnormalities detected in micro- and megasporogenesis in Ranunculus. Table S2: More detailed information on the generalized mixed-effect model (GLMM) analyses. These analyses observed effects changing the error frequency of micro- and megasporogenesis in Ranunculus with regard to ploidy, generation and sex. Calculations were based on 115 Ranunculus plants and more than 13,000 individual data points. R calculation output is visualized including standard error and z value. Regression estimate and p value are calculated by GLMM analysis and the tested factor is referred to the test and base line categories. Table S3: Mean peak indices of reproductive mode of different Ranunculus populations. Fig. S1: Asexual ES formation in an ovule of a diploid Ranunculus F2 hybrid (taken from [40]). Fig. S2: Chi-squared analyses of erroneous mega- and microsporogenesis in natural and hybrid Ranunculus plants. Fig. S3: Representative flow cytometry histograms of Ranunculus seeds. [file 12870_2020_2654_MOESM1_ESM.zip › Suppl. Table S2_former Suppl. Table S1.doc]

Table S2: Generalized mixed-effect model (GLMM) analyses observing effects changing the error frequency of micro- and megasporogenesis in Ranunculus with regard to ploidy level, generation and sex. Calculations were based on 115 Ranunculus plants and more than 13,000 individual data points. R calculation output is visualized including standard error and z value. Regression estimate and p value are calculated by GLMM analysis and the tested factor is referred to the test and base line categories.

| Subset | n | tested factor(s) | base line | test category | GLMM Regression Estimate | Standard Error | z value | p value |
| --- | --- | --- | --- | --- | --- | --- | --- | --- |
| Male | 9193 | ploidy level |  | (intercept) | -3.99 | 0.13 | -31.85 | < 0.001 |
|  |  |  | 2*x* | 4*x* | 2.19 | 0.23 | 9.38 | < 0.001 |
|  |  | generation | F_2_ | P | -0.77 | 0.33 | -2.35 | < 0.05 |
|  |  |  | F_2_ | F_1_ | -0.63 | 0.22 | -2.85 | < 0.01 |
|  |  | combined  effect | 2*x*, F_2_ | 4*x*, P | -0.88 | 0.50 | -1.76 | 0.08 |
|  |  |  | F_2_ | 4*x*, F_1_ | -0.60 | 0.35 | -1.71 | 0.09 |
| Female | 3660 | ploidy level | 2*x* | (Intercept) | -1.55 | 0.04 | -35.08 | < 0.001 |
|  |  |  |  | 4*x* | 0.17 | 0.24 | 0.73 | 0.46 |
| Male/  Female | 7438 | ploidy level |  | (Intercept) | -1.55 | 0.04 | -35.08 | < 0.001 |
|  |  |  | 2*x* | 4*x* | 0.17 | 0.24 | 0.73 | 0.46 |
|  |  | sex | female | male | -2.44 | 0.13 | -18.36 | < 0.001 |
|  |  | combined  effect | 2*x*, female | 4*x*, male | 2.02 | 0.33 | 6.05 | < 0.001 |
