# Supplementary material for: The relation of meiotic behaviour to hybridity, polyploidy and apomixis in the Ranunculus auricomus complex (Ranunculaceae)
Source: BMC Plant Biol. 2020 Nov 17;20:523. doi: 10.1186/s12870-020-02654-3 (PMC7672892; doi:10.1186/s12870-020-02654-3)
Supplement: Supplementary file 1 — Additional file 1: Table S1: Number of cytological abnormalities detected in micro- and megasporogenesis in Ranunculus. Table S2: More detailed information on the generalized mixed-effect model (GLMM) analyses. These analyses observed effects changing the error frequency of micro- and megasporogenesis in Ranunculus with regard to ploidy, generation and sex. Calculations were based on 115 Ranunculus plants and more than 13,000 individual data points. R calculation output is visualized including standard error and z value. Regression estimate and p value are calculated by GLMM analysis and the tested factor is referred to the test and base line categories. Table S3: Mean peak indices of reproductive mode of different Ranunculus populations. Fig. S1: Asexual ES formation in an ovule of a diploid Ranunculus F2 hybrid (taken from [40]). Fig. S2: Chi-squared analyses of erroneous mega- and microsporogenesis in natural and hybrid Ranunculus plants. Fig. S3: Representative flow cytometry histograms of Ranunculus seeds. [file 12870_2020_2654_MOESM1_ESM.zip › Suppl. Table S3_former Suppl. Table S2.doc]

Table S3: Mean peak indices (PI) of reproductive mode of different Ranunculus populations. The peak index (endosperm DNA content/ embryo DNA content) indicates a seed formed by sexual double fertilization, resulting in a 2n embryo and a 3n endosperm.

| Population | Species | Ploidy Level | No. of Seeds | PI (Range) | Reproduction Mode |
| --- | --- | --- | --- | --- | --- |
| LH008 | *R. cassubicifolius* | 4*x* | 13 | 1.51 (1.46 - 1.56) | sexual |
| LH009 | *R. cassubicifolius* | 4*x* | 12 | 1.46 (1.34 - 1.51) | sexual |
| LH040 | *R. carpaticola* | 2*x* | 14 | 1.53 (1.44 - 1.60) | sexual |
